# Assessment of left ventricular regional function in affected and carrier dogs with duchenne muscular dystrophy using speckle tracking echocardiography

**DOI:** 10.1186/1471-2261-11-23

**Published:** 2011-05-25

**Authors:** Hiroshi Takano, Yoko Fujii, Naoko Yugeta, Shinichi Takeda, Yoshito Wakao

**Affiliations:** 1 Department of Surgery 1, School of Veterinary Medicine, Azabu University, Kanagawa, Japan; 2 Department of Molecular Therapy, National Institute of Neuroscience, National Center of Neurology and Psychiatry, Tokyo, Japan

## Abstract

**Background:**

Two-dimensional speckle tracking echocardiography (STE) is a relatively new method to detect regional myocardial dysfunction. To assess left ventricular (LV) regional myocardial dysfunction using STE in Duchenne muscular dystrophy model dogs (CXMD_J_) without overt clinical signs of heart failure.

**Methods:**

Six affected dogs, 8 carrier dogs with CXMD_J_, and 8 control dogs were used. Conventional echocardiography, systolic and diastolic function by Doppler echocardiography, tissue Doppler imaging (TDI), and strain indices using STE, were assessed and compared among the 3 groups.

**Results:**

Significant differences were seen in body weight, transmitral E wave and E' wave derived from TDI among the 3 groups. Although no significant difference was observed in any global strain indices, in segmental analysis, the peak radial strain rate during early diastole in posterior segment at chordae the tendineae level showed significant differences among the 3 groups.

**Conclusions:**

The myocardial strain rate by STE served to detect the impaired cardiac diastolic function in CXMD_J _without any obvious LV dilation or clinical signs. The radial strain rate may be a useful parameter to detect early myocardial impairment in CXMD_J_.

## Background

Duchenne progressive muscular dystrophy (DMD) is characterized by progressive degeneration of skeletal and cardiac muscles with fibrotic tissue replacement and fatty infiltration[1]. Resulting myocardial dysfunction has been estimated to be responsible for death in 20% of human DMD patients[2,3].

Echocardiography is one of the useful noninvasive methods used to assess cardiac function in patients and animals with Duchenne's cardiomyopathy[4-9]. It is possible that the recognition of earlier subclinical cardiac systolic or diastolic dysfunction could allow for an early medical approach, thus improving long-term cardiovascular outcomes. Several studies have demonstrated the usefulness of tissue Doppler imaging (TDI) to detect subclinical myocardial systolic and diastolic dysfunction in patients with normal conventional echocardiographic parameters[10]. These studies have also confirmed that myocardial velocities, myocardial wall-thickening velocities, myocardial velocity gradients and strain during systole and early diastole in the left ventricular free wall were reduced in patients[11-15] and dogs with DMD[16,17]. However, strain measurements derived from TDI involve several disadvantages including angle-dependence and the limitations of an available cardiac region for an assessment. Since it has been reported that the distribution of myocardial lesions detected by magnetic resonance imaging (MRI) and single photon emission computed tomography (SPECT) varied among DMD patients[18-20], TDI might underestimate the severity of myocardial dysfunction in certain patients.

Two-dimensional speckle tracking echocardiography (STE) is a new approach designed to assess left ventricular function in humans and animals[21,22]. STE has no angle-dependence and allows the assessment of any region of the heart. Usefulness of STE analysis in human patients with idiopathic dilated cardiomyopathy to detect systolic and diastolic dysfunction has been described in several reports. STE provides several advantages over TDI, in for example, estimation for the increase in LV filling pressure[23-25]. In addition, STE has the ability to detect regional myocardial dysfunction[22]. We hypothesized that STE could detect early myocardial lesions in patients with Duchenne's cardiomyopathy without clinical signs. To our knowledge, there have been no reports assessing the cardiac function of Duchenne's cardiomyopathy using STE.

Canine X-linked muscular dystrophy in Japan (CXMD_J_) is a Beagle-based dog colony established by artificially inseminating frozen semen from spontaneous Golden retriever muscular dystrophy. CXMD_J _has been reported to be comparable to human patients and dogs with DMD[9,26-28]. The purpose of the present study was to assess left ventricular regional myocardial dysfunction using STE in affected and carrier CXMD_J _dogs without overt clinical signs of heart failure.

## Methods

### Animals

Beagles aged 8 months old or more from a CXMD_J _colony at the Division of Laboratory Animal Resources, National Institute of Neuroscience, National Center of Neurology and Psychiatry used in the present study included 6 dogs affected with CXMD_J_, 8 carrier dogs, and 8 control dogs. Control dogs had no history of cardiopulmonary diseases, and each had a normal physical examination, standard 6 lead electrocardiogram and conventional echocardiogram. Dogs were categorized as affected, carrier or normal (control) dogs on the basis of DNA analysis conducted immediately after the birth[27]. All experiments were approved by the Ethics Committee for the Treatment of Laboratory Animals of the National Institute of Neuroscience (approval No. 20-03 and 21-03).

### Echocardiographic Examination

All echocardiographic images were acquired using Vivid S6 (GE Medical System, Tokyo, Japan) ultrasound unit equipped with a 7 MHz transducer and were obtained by one trained examiner (HT). Dogs were restrained manually in lateral recumbency. Skilled experimental animal technicians handled the dogs and assisted in the experiments. For dogs that became agitated, the examination was performed 15 minutes after sedation with acepromazine (0.01 mg/kg, IV, A.C.P. 10, 10 mg/mL, Delvet, The State of New South Wales, Australia) and buprenorphine (0.0075 mg/kg, IV, Lepetan injection, 0.2 mg/mL, Otsuka, Tokyo, Japan). ECG monitoring with clear R wave recognition was recorded in concurrence with an echocardiographic examination using the same ultrasound unit. The mean value of variables in 3 consecutive cardiac cycles was used for statistical analysis.

### Conventional Echocardiography

M-mode echocardiographic measurements were made from the right parasternal short-axis view at the chordae tendineae (Ct) level (LV end-diastolic and end-systolic internal diameters [LVIDd and LVIDs], and fractional shortening [FS]). We calculated left ventricular end-diastolic volume (EDV), left ventricular end-systolic volume (ESV) and left ventricular ejection fraction (EF) from LV internal diameters using the Teichholz method. LV end-diastolic and end-systolic volume indices (EDVI and ESVI) were derived from EDV and ESV divided by body surface area (BSA), respectively[29]. Diameters of the left atrium (LAD) and aortic root (AoD) were measured from the right parasternal short-axis view at the heart base level by B-mode method, and the LA/Ao ratio was calculated[30].

### Systolic and Diastolic Function by Doppler Echocardiography and TDI

Systolic time intervals (pre-ejection period [PEP] and LV ejection time [ET]) were measured using an aortic flow velocity curve[31]. Transmitral inflows (TMF) were created from the left parasternal apical 4-chamber view. Early (E wave) and late (A wave) filling velocities, the E wave deceleration time (DcT) and E/A ratio were measured from the transmitral flow tracing. Left ventricular isovolumic relaxation time (IVRT) was calculated as the time difference between the intervals from the beginning of the Q wave on the ECG to the onset of early diastolic flow and intervals from the Q wave to the end of aortic flow tracing. Tissue Doppler trace of mitral annulus velocity (MAV) on the lateral side was obtained from the left parasternal apical 4-chamber view to measure peak velocities during systole (S') and during early diastole (E'). Then, the E/E' was calculated[32,33].

### Speckle Tracking Echocardiography (STE)

Right parasternal short-axis views at Ct level were used to measure all STE indices below, because myocardial lesion of dogs with CXMD_J _was reported to be localized in free wall at basal level of LV[9]. Images were acquired in cine loops triggered by the QRS complex, saved in digital format, and analyzed using off-line software (EchoPAC PC '08 ^a^). The principal of speckle tracking analysis has been described in several previous studies[21,22,34-37]. All analysis was performed by one observer (HT).

**Figure 1:**
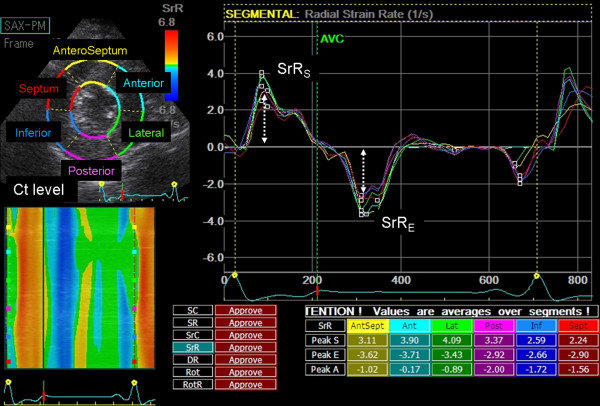
**Radial strain rate tracking for one cardiac cycle obtained from the parasternal short-axis view at Ct level**. Six curves with different colors depict respective each myocardial segments of left ventricule (anteroseptum, anterior, lateral, posterior, inferior and septum). Systolic and early diastolic values of radial strain rate (SrR_S _and SrR_E_) were calculated.

For the each of 6 regions of interest, peak systolic radial and circumferential strains (SR and SC), and peak radial and circumferential strain rates during systole and early diastole (SrR_S_, SrR_E_, SrC_S _and SrC_E_) were measured at the level of the Ct (Figure 1). The E/SrR_E _and E/SrC_E _were derived from E divided by SrR_E _and SrC_E_, respectively. Since the values of radial and circumferential direction were calculated from 6 segments as described previously, the mean of all 6 segments and the mean of each segmental value were calculated for all parameters.

### Experimental Protocols

Conventional echocardiographic parameters, systolic time intervals, diastolic functional parameters and STE indices were obtained and compared among the affected, carrier and normal dogs.

Since sedatives were administered when a dog was not cooperative, the influence of sedation on all indices was assessed using 6 normal dogs. Parameters were obtained at baseline and 15 minutes after sedation with acepromazine (0.01 mg/kg, IV) and buprenorphine (0.0075 mg/kg, IV), and then compared.

For the assessment of reproducibility, intraobsever variability of STE analysis was assessed by use of images from 6 normal dogs. Using same cine loops, each STE value was determined again 3 weeks after the primary analysis.

### Statistics

Comparisons among the 3 groups were performed using one-way ANOVA. A post hoc testing for the difference among the groups was performed by the Steel method. The assessment of the influence of sedation was performed using the paired *t*-test. When the normality test failed, the Wilcoxon signed-rank test was applied. Intraobserver variability was express as mean of absolute difference as the percentage of the mean of two absolute measurements. A value of *P *< 0.05 was considered statistically significant.

## Results

### Animals

The characteristics of affected, carrier and normal dogs in the present study are shown in Table 1. There was a significant difference in BW among the 3 groups.

**Table 1:** Characteristics of each group

Index	Unit	Normal dogs	Carrier dogs	Affected dogs
Number		8	8	6
Gender	F/M	4/4	8/0	3/3
Number of sedated dogs		5	6	4
Age	months	32.86 ± 29.73	33.13 ± 23.68	24.50 ± 15.27
Body weight	kg	12.19 ± 1.04	11.41 ± 1.22	9.49 ± 2.05†

†: Significant difference compared with normal dogs by multiple comparison (*P *< 0.05).

### Influence of Sedation

A significant difference between pre- and post-sedation values was seen in HR and ESVI. In addition, a significant difference was also seen in the A wave of TMF (Table 2).

**Table 2:** Effects of sedation on echocardiographic parameters.

Index		Unit	Awake	Sedated	*P *value
Conventional echocardiography	HR	bpm	89.10 ± 27.29	69.61 ± 7.01	< 0.05
	ESVI	mL/m^2^	27.37 ± 3.64	21.43 ± 3.61	0.0042

Transmitral flow	A wave	cm/sec	46.33 ± 12.41	34.22 ± 8.28	0.015

(results with *P *value < 0.05)

ESVI = Left ventricular end-systolic index; A = Peak velocity of late diastolic flow in transmitral flow.

### Conventional Echocardiography

No significant difference among the 3 groups was noted in any parameters (Table 3). Mild mitral regurgitation (MR) was detected using the color Doppler imaging in 1 carrier dog.

**Table 3:** Conventional echocardiographic parameters of each group

Index	Unit	Normal dogs	Carrier dogs	Affected dogs
HR	bpm	92.54 ± 11.22	106.39 ± 26.22	102.16 ± 35.79
EF	%	65.42 ± 8.13	67.13 ± 10.35	62.89 ± 13.05
FS	%	35.04 ± 6.66	36.92 ± 8.41	33.61 ± 9.87

EDVI	mL/m^2^	59.19 ± 19.53	69.79 ± 20.62	53.35 ± 27.03
ESVI	mL/m^2^	15.43 ± 4.57	20.04 ± 11.02	17.87 ± 13.75

LA/Ao		1.22 ± 0.095	1.17 ± 0.14	1.08 ± 0.081

EF = Ejection fraction; FS = Fractional shortening; EVDI = Left ventricular end-diastolic index; ESVI = Left ventricular end-systolic index; LA/Ao = Left atrial diameter/aortic root diameter ratio.

### Systolic and Diastolic Function by Doppler Echocardiography (Table 4)

**Table 4:** Echocardiographic systolic and diastolic parameters using Doppler of each group

Index		Unit	Normal dogs	Carrier dogs	Affected dogs
Systolic time intervals	PEP/ET		0.21 ± 0.037	0.21 ± 0.046	0.22 ± 0.085

Transmitral flow	E wave	cm/sec	74.71 ± 13.42	87.42 ± 10.89†	66.44 ± 10.19
	A wave	cm/sec	45.79 ± 14.68	45.71 ± 8.09	44.61 ± 8.98
	E/A		1.82 ± 0.71	1.98 ± 0.44	1.58 ± 0.52
	DcT	msec	92.29 ± 8.20	91.54 ± 14.44	91.06 ± 14.73

Isovolumic relaxation time		msec	32.13 ± 11.49	28.21 ± 10.07	39.28 ± 13.61

Mitral annular velocity	S' wave	cm/sec	10.67 ± 1.60	12.17 ± 5.94	7.39 ± 1.25
	E' wave	cm/sec	12.13 ± 2.62	11.33 ± 2.12	8.67 ± 1.65†
	E/E'		6.39 ± 1.57	7.89 ± 1.41	8.48 ± 2.17

†: Significant difference compared with normal dogs by multiple comparison (*P *< 0.05).

PEP = Pre-ejection period; ET = Left ventricular ejection time; E = Peak velocity of early diastolic flow in transmitral flow; A = Peak velocity of late diastolic flow in transmitral flow; DcT = Deceleration time of early diastolic flow; S'= Peak mitral annular velocity during systole; E'= Peak mitral annular velocity during early diastole.

Significant differences were seen in E wave of TMF and MAV on the lateral side (E') among the 3 groups.

### Speckle Tracking Echocardiography

The frame rate used for this study to analyze STE indices ranged from 72 to 93 frames per second. Frames per cardiac cycle were 57.38 ± 6.53, 49.36 ± 10.70 and 49.44 ± 12.16 frames, in normal, carrier and affected dogs, respectively, and there was no significant difference among the 3 groups (*P *= 0.21). Intraobserver variability of measurements was 3.6 to 13.2%. Table 5 shows the results of global values in the STE indices of the 3 groups. In strain indices, only E/SrR_E _had significant differences between normal and carrier dogs.

**Table 5:** STE indices of each group

Index		Unit	Normal dogs	Carrier dogs	Affected dogs
Radial	SR	%	44.50 ± 9.63	49.58 ± 14.76	46.68 ± 12.63
	SrR_S_	/sec	2.71 ± 0.41	2.82 ± 0.68	2.78 ± 0.63
	SrR_E_	/sec	-3.06 ± 0.50	-2.55 ± 0.73	-2.23 ± 0.95
	E/SrR_E_		-24.81 ± 5.21	-36.20 ± 8.73†	-34.24 ± 12.88

Circumferential	SC	%	-20.87 ± 3.86	-22.76 ± 5.14	-23.04 ± 5.38
	SrC_S_	/sec	-2.49 ± 0.26	-2.77 ± 0.72	-3.04 ± 0.86
	SrC_E_	/sec	2.84 ± 0.61	2.92 ± 0.78	2.70 ± 0.49
	E/SrC_E_		27.15 ± 7.08	31.80 ± 8.92	25.50 ± 6.48

†: Significant difference compared with normal dogs by multiple comparison (*P *< 0.05).

SR = peak systolic radial strain; SrR_S _= Peak radial strain rate during systole; SrR_E _= Peak radial strain rate during early diastole; SC = Peak systolic circumferential strain; SrC_S _= Peak circumferential strain rate during systole; SrC_E _= Peak circumferential strain rate during early diastole.

**Figure 2:**
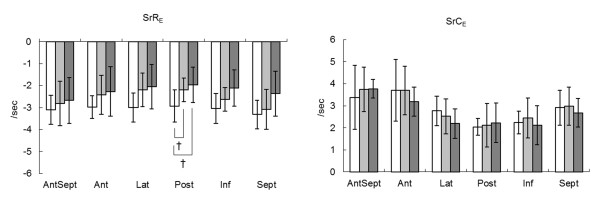
**Segmental values of radial and circumferential strain rates during early diastole at apical and Ct levels in affected (black bars), carrier (gray bars), and normal (white bars) dogs**. Data were shown as mean ± SD. †: Significant difference compared with normal dogs by multiple comparisons (*P *< 0.05). SrR_E _= Peak radial strain rate during early diastole, SrC_E _= Peak circumferential strain rate during early diastole, AntSept = anteroseptum, Ant = anterior, Lat = lateral, Post = posterior, Inf = inferior, Sept = septum

SrR_E _in the posterior segments were significantly decreased in carrier and affected dogs when segmental values were compared with normal dogs (Figure 2 and table 6).

**Table 6:** Segmental values of peak radial strain rate during early diastole at Ct level in each group

Segment	Unit	Normal dogs	Carrier dogs	Affected dogs
Anterioseptum	/sec	-3.10 ± 0.66	-2.81 ± 1.01	-2.68 ± 1.05
Anterior	/sec	-2.98 ± 0.51	-2.43 ± 0.89	-2.27 ± 1.13
Lateral	/sec	-3.00 ± 0.66	-2.19 ± 0.76	-2.04 ± 1.01
Posterior	/sec	-2.93 ± 0.73	-2.19 ± 0.54†	-1.96 ± 0.81†
Inferior	/sec	-3.04 ± 0.68	-2.63 ± 0.53	-2.10 ± 0.83
Septum	/sec	-3.32 ± 0.64	-3.08 ± 0.91	-2.36 ± 1.03

†: Significant difference compared with normal dogs by multiple comparison (*P *< 0.05).

## Discussion

In the present study, no significant difference was found in any parameters using conventional echocardiography among the 3 groups, which indicated no apparent LV dilation or systolic dysfunction with normal FS in affected and carrier dogs.

SrR_E _has been reported to show a significant correlation with LV relaxation[38,39]. A significant difference in SrR_E _was observed in posterior segments at the Ct level in carrier and affected dogs. SrR_E _tended to decrease in other LV segments. The results of segmental STE analysis suggest an impairment of LV relaxation in the basal inferoposterior segments of CXMD_J _- affected dogs prior to a detectable decrease in global indices of cardiac function. In previous studies, pathological lesions were frequently observed in the posterior and lateral wall of the left ventricle in patients and dogs with DMD[9,40,41]. Previous reports have indicated that TDI-derived parameters also identified myocardial dysfunction in the LV free wall[11,14-17,42], which supported our results.

Circumferential strain parameters in affected dogs were not significantly changed in the present study. In contrast, Mertens et al. reported significant decreases in TDI-derived strain indices in the apical, mid and basal level of LV long-axis views in DMD patients[15]. One possible explanation for this difference is more advanced disease in the previously reported population, supported by the presence of obvious LV dilation in the patients of that study. A second possibility is different measurement techniques. Mizuguchi et al. have suggested that LV systolic dysfunction may begin with reduced longitudinal shortening that is compensated by augmented circumferential shortening in early stages. Therefore, one could hypothesize that STE performed using long-axis views is a more sensitive method than with short-axis views, although it was not evaluated in the present study.

E wave velocity in carrier dogs was significantly increased compared with normal dogs. Increased E wave was noticed in dogs with overt and occult dilated cardiomyopathy (DCM), which may suggest diastolic dysfunction[43]. However, in the present study, the other measured parameters derived from transmitral flow, such as DcT and A wave velocity, were all within the reference range and no statistical significance was found among groups. In addition, the TMF pattern was not the typical restrictive pattern (i.e. E/A>2 as well as short DcT) in any individual carrier dogs[43,44]. The clinical relevance of this finding is uncertain given the relatively small magnitude of difference and the large amount of overlap in values among groups. In addition, higher value of E/SrR_E _in carrier dogs might be influenced by the increased E wave. E' measured using TDI was significantly reduced in affected dogs compared with normal dogs. This is similar to a previous report of reduced peak E' velocity in patients with DMD[13].

Duchenne's cardiomyopathy in a carrier female was previously reported, though it was found to be mild compared with that in an affected male[45,46]. In the present study, there was significant difference in segmental assessment of SrR_E _between normal and carrier female dogs as well as affected dogs. Since our carrier females were young to middle-aged, it is possible that follow-up evaluation would reveal myocardial impairment.

In the present study, low dose of acepromazine was used for sedation. The influence of sedation was seen in EDVI, and A wave velocity of TMF. Schaefer et al. demonstrated increased A waves of TMF and myocardial velocity using TDI under anesthesia in normal mice, while increased heart rates were observed at the same time[47]. They speculated that changes in heart rates constituted one of the factors causing increased A wave velocity. Since changes in A waves in our study were accompanied by decreased heart rates, there might have been a relationship. Although further study is needed, parameters influenced by sedation appeared to be less important in the overall interpretation of our results. Additionally, proportions of sedated dogs in each group were similar.

There were several limitations in the present study. First, the number of dogs available for each group was limited and age distribution was relatively wide in all groups because of the difficulty in maintaining a sufficient number of CXMD_J_s. Second, body weight in affected dogs was significantly lower than in normal dogs. However, we consider that this difference would be less likely to affect our results. Third, one carrier dog had mild MR. A report described mitral valve prolapse in DMD patients, and MR was considered to be responsible for LV dilation[48]. In the present study, because LV dilation was not observed in that carrier dog, MR was more likely caused by myxomatous degeneration of the mitral valve, the most common acquired heart disease in dogs. In this case, MR was considered to be too mild to likely affect our results. Fourth, the accuracy of strain measurement depends on the quality of 2-dimensional echocardiography and frame rate. The mean frame rates per cardiac cycle in normal, carrier and affected dogs were 57.4, 49.4 and 45.1, respectively. The frame rate per cardiac cycle was relatively low due to the relatively higher heart rate compared with humans (about 70-110 frames when the human heart rate was considered to be 60 bpm [49]). This may affect the accuracy of tracking quality, especially in early diastolic parameters. Further investigation using newer methods might detect change more precisely[50]. Finally, given the characteristics of this disease, differences in the sex ratio among the groups were unavoidable. Since significant differences between males and females in some STE parameters have been reported in a human study[51], the results could have been influenced by gender. Since the number of normal dogs was small when categorized by gender, further studies using a greater number of dogs are warranted. In addition, there should be some differences in expression of the disease among the affected dogs, and CXMDJ might have less severe disease compared to the human patients.

## Conclusions

The myocardial strain rate by STE served to detect the impaired cardiac diastolic function in CXMD_J _without any obvious LV dilation or clinical signs. The radial strain rate may be a useful parameter to detect early myocardial impairment in CXMD_J_.

## List of abbreviations

LV: left ventricular; STE: speckle tracking echocardiography; CXMDJ: canine X-linked muscular dystrophy; TDI: tissue Doppler imaging; DMD: Duchenne's muscular dystrophy; Ct: chordae tendineae; LVIDd: left ventricular end-diastolic internal diameter; LVIDs: left ventricular end-systolic internal diameter; FS: fractional shortening; EF: ejection fraction; EDVI: end-diastolic volume index; ESVI: end-systolic volume index; EDV: end-diastolic volume; ESV: end-systolic volume; BSA: body surface area; LAD: left atrial diameter; AoD: aortic root diameter; LA/Ao: left atrium to aorta ratio; PEP: pre-ejection period; ET: ejection time; TMF: transmitral flow; DcT: deceleration time; IVRT: isovolumic relaxation time; MAV: mitral annulus velocity; SR: peak systolic radial strain; SC: peak systolic circumferential strain; SrR_S_: peak radial strain rate during systole; SrR_E_: peak radial strain rate during early diastole; SrC_S_: peak circumferential strain rate during systole; SrC_E_: peak circumferential strain rate during early diastole; MR: mitral regurgitation

## Competing interests

The authors declare that they have no competing interests.

## Authors' contributions

HT performed echocardiographic examinations for all dogs and statistical analysis. NY and ST contributed to hold this genetic colony of CXMDJ, and carried out genetic examinations to determine each dog's genetic status. YF and YW conceived of the study, and participated in its design and coordination. All authors read and approved the final manuscript.

## Pre-publication history

The pre-publication history for this paper can be accessed here:

http://www.biomedcentral.com/1471-2261/11/23/prepub
